# Agreement between simulated patients and faculty: Assessment of communication skills during objective structured clinical examination

**DOI:** 10.12669/pjms.35.6.1000

**Published:** 2019

**Authors:** Farheen Yousuf, Naveed Yousuf

**Affiliations:** 1 Dr. Farheen Yousuf, MRCOG, FCPS. Department of Obstetrics and Gynecology, Medical College, Aga Khan University, Karachi, Pakistan; 2 Dr. Naveed Yousuf, PhD. Department for Educational Development, Faculty of Health Sciences, Aga Khan University, Karachi, Pakistan

**Keywords:** Communication and counseling skills, Inter-rater reliability, OSCE, Simulated-patients

## Abstract

**Objective:**

Ensuring competence in communication skills amongst trainees is essential in health professions education. Involving faculty members for the same is a challenge in Obstetrics and Gynecology (OBGYN) due to their clinical commitments. The present study compares scores of OBGYN faculty, non-OBGYN faculty and simulated patients (SPs) on communication skills of postgraduate trainees during formative Objective Structured Clinical Examination (OSCE).

**Methods:**

This is a psychometric study conducted in Feburary 2017 at the Aga Khan University Medical College (AKU-MC). All thirty-two postgraduate trainees of OBGYN gave consent. Each trainee was assessed by OBGYN faculty, non-OBGYN faculty and SP on communication skills at six stations using nine-point itemized rating-scale during formative OBGYN OSCE. The scores were reviewed using descriptive statistics, reliability was calculated using Cronbach’s alpha and inter-rater reliability was analyzed using Pearson correlation and intra-class correlation coefficient.

**Results:**

The score reliability of each of the examiners was >0.7. The mean scores showed that OBGYN faculty were most stringent while SPs were lenient examiners, however, non-OBGYN faculty scored in between. The inter-rater reliability among any two of the OBGYN, non-OBGYN and SP examiner was >0.84 using Pearson correlation and >0.9 using intra-class correlation.

**Conclusion:**

The SPs and non-OBGYN clinical faculty can also be used to assess communication and counseling skills on OBGYN OSCEs after required training as examiners.

## INTRODUCTION

Communication skill is one of the essential competency in health professions.^1^ This is an integral part of the graduate curriculum in the Accreditation Council of Graduate Medical Education, General Medical Council and Liaison Committee on Medical Education.^2^-^4^ The College of Physicians and Surgeons Pakistan also include communication skill as one of the fundamental competency in postgraduate training.^5^ Ineffective communication among patients and doctors may end up in confusion that further leads to medical errors and complications.^6^ Therefore, interpersonal and communication skills are the international patient safety goals for Joint Commission International Accreditation.^7^ The students also recognize communication skill as an essential skill that should be taught during training.^8^

The training programs on communication skills are scarce, lack of systematic guidance and standardization amid limited clinical opportunities leads to deficient health practices.^8^ The mainstay of communication skills training providers are social scientists, general practitioners and medical educators, however they also have no or minimal training in this domain.^9^ It is well recognized that teaching sessions on communication skills with simulated patients (SPs) increases the scores on as shown in post-test assessment.^8^ Use of SPs gives the opportunity to practice and rehearse difficult and challenging situations and help to improve critical thinking and self-confidence.^8^,^10^,^11^ Simulated patients can also be utilized to portray themselves as patients in Objective Structured Clinical Examinations (OSCE) or other validated assessment of communication and counselling skills.^12^ Nonetheless a mixed method study conducted in Pakistan, found no difference in the scores of fourth year medical students whether real or simulated patients were used for interaction during the examination.^13^ The students choose simulated patients in place of real patients to be used in exam setting for assessment of communication skills and 97% of students found SPs provide motivation because it is not difficult to deal with them.^13^

Ensuring competence in communication skills amongst trainees through assessments is equally essential in health professions education.^5^ Obstetrics and Gynecology is having highest rate of litigations among all medical professions.^14^ Involving clinical faculty for assessments, however, is a challenge. All clinicians are thriving with busy clinical practices, struggling with clinical, research and educational work. This is also true for faculty in Obstetrics and Gynecology (OBGYN) department. Keeping them engaged in OSCEs is further challenging. One way to overcome this challenge is to engage SPs as examiners for communication skills in OSCEs. SPs being layman with minimum medical knowledge may assess the communication skills after training. Other option would be including faculty outside OBGYN (non-OBGYN) as examiners for communication skills in OBGYN.

A study conducted in Pakistan in 2012 used simulated patient to assess the communication and interpersonal skills of Radiology residents showed correlation between 0.3-0.5 among faculty and SPs.They found that the SP were not fully equipped with regard to training and experience.^15^ A Canadian study depict that students felt that SPs as examiners are less stressful and competent to judge their clinical examination skills.^16^

The primary objective of this study was to investigate the inter-rater reliability of scores between OBGYN faculty, non-OBGYN faculty and SPs for assessment of communication and counselling skills during OSCE in OBGYN.The secondary objective was to determine the reliability of scores using OBGYN faculty, non-OBGYN faculty and SP as assessors for assessment of communication and counselling skills during OSCE in OBGYN.

## METHODS

This is a psychometric study conducted in Feburary 2017 at the Aga Khan University Medical College (AKU-MC). A formative twelve station OSCE examination was organised for the post-graduate trainees in OBGYN. Six of these twelve stations were based on assessment of communication and counseling skills that are included in the present study. These were based on commonly encountered scenarios faced by a physician in OBGYN such as; breaking bad news to a lady whose baby was still birth during cesarean section, revealing a report to the patient with malignancy, taking informed consent before hysterectomy, counselling of patients with gestational diabetes and vaginal birth after cesarean section along with dealing with angry patient whose surgery was delayed due to some other emergency case. Each station was assessed by three examiners: simulated patient, OBGYN faculty and non-OBGYN faculty.

### Assessment Tool

Patient-centered observation form (PCOF) was used for scoring having reliability of 0.67. It is user friendly and relevant to our context and community expectations.^6^,^9^ Permission was obtained to use PCOF with minor changes in our study. This form was also translated in Urdu for better understanding of SP’s. We used the nine-point rating scale adapted from MiniCEX to be used for rating of trainees’ performance on the PCOF items.^17^

### Selection and Training of Simulated Patients (SPs)

Six female SP’s were recruited for the present study with their consent from the SP bank at AKU-MC based on the age, qualifications and years of experience as required. The SPs were trained during a three-hour session on the OSCE scenarios and to use PCOF. To ensure that the SPs understood the use of PCOF for assessment of communication skills, a one-on-one briefing session was held with each SP by the training faculty. Later, each SP demonstrated the scenario with the faculty member in front of the other five SPs. All other SPs were asked to rate the SP faculty interaction using the PCOF form. These ratings were then discussed to ensure standardization of assessment by SPs.

### Selection and Training of Faculty

Six OBGYN and six non-OBGYN faculty members, who volunteered to take part in this activity were invited as examiners. They were all experienced and actively involved in conducting OSCE examinations as assessors. PCOF was shared via email followed by one-hour training to use the PCOF form for assessment of communication skills.

### Selection of Trainees

All OBGYN trainees registered for Fellow of College of Physicians and Surgeons Pakistan (FCPS) Part-2 and Member of College of Physicians and Surgeons Pakistan (MCPS) working at AKU-MC and at a secondary hospital campus were invited through email for this formative OSCE. Thirty-two trainees consented to participate in this study. Their age along with years of experience after graduation (MBBS) were noted.

### Ethical Review

Ethical approval was obtained from Ethical Review Committee and OBGYN Department at AKU and The Research and Training Monitoring Cell (RTMC) of the College of Physician and Surgeon, Karachi, Pakistan. (3876-Obs-ERC-15)

### Statistical analysis

All data was entered in SPSS 20 for analysis. Descriptive statistics were computed for scores by each assessor, that is, OBGYN faculty, non-OBGYN faculty and SPs. The reliability (internal consistency) of scores by each examiner were analyzed using Cronbach’s alpha. The inter-rater reliability was analyzed through Pearson’s correlation and intra-class correlation coefficient.

## RESULTS

All 32 trainees appeared in the OSCE. Amongst them, twenty-five were FCPS part-2 trainees while remaining were MCPS trainees. Their mean age was 26 years (SD±2). Their clinical experiences ranged between one to five years.The trainees were assessed by three examiners on each station, that is, OBGYN faculty, non-OBGYN faculty and an SP.

The six OBGYN examiners were from the department of Obstetrics and Gynecology at AKU-MC. The non-OBGYN faculty members included three anesthetists, two general surgeons and one medical educationist with a clinical background. All faculty members had an experience of more than five years as OSCE examiners.

All SPs had an experience of more than six years of being involved in OSCEs. Four of them had a Bachelors qualification, while the other two had twelve-years of education. The overall reliability of scores on the six communication skills stations for each of the three examiners were >0.7. (Table-I).

**Table I:** Reliability of assessors and their mean scores.

Examiner	Reliability (Cronbach’s alpha)	Mean ± SD	Minimum	Maximum
OBGYN faculty	0.749	49.2 ±3.92	45.06	56.09
Non-OBGYN faculty	0.782	53.7 ±4.87	45.12	57.71
SP	0.777	65.2 ±11.72	51.08	78.93

**Fig. 1:**
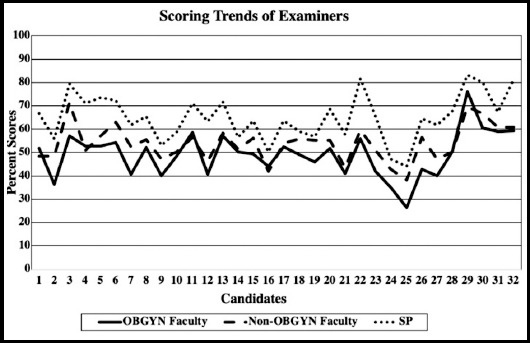
Scoring trends of the examiners.

The mean and standard deviation of communication skills scores over the six stations for OBGYN faculty, non-OBGYN faculty and SP are also shown in Table-I. The mean scores showed that OBGYN faculty (subject experts) were the most stringent while the SPs were the most lenient examiners, however non-OBGYN faculty members scored in between the two. Further exploration of the scoring trend, for each of the trainees by each of the three examiners, showed a very similar trend (Fig. 1).

For inter-rater reliability, the Pearson correlation and Intra-class correlation coefficient are shown in Table-II. The Pearson correlation of scores was >0.8 between any two of the three examiners, and likewise the intra-class correlation coefficient was >0.9.

**Table II:** Inter-rater agreement between the three examiners.

	Pearson correlation	Intra-class correlation
OBGYN & non-OBGYN	0.84	0.903
OBGYN & SP	0.84	0.914
Non-OBGYN & SP	0.85	0.902

## DISCUSSION

Our study compared faculty and simulated patients (SPs) scores on communication skills of postgraduate trainees during formative OSCE. This study showed good reliability of the OSCE scores by all examiners ranging 0.7-0.8 that included simulated patients. The inter-rater reliability of any of the two examiners (OBGYN faculty, non-OBGYN faculty and SP) was also high (0.8-0.9).

The present study showed that if SPs are well trained they can be used as assessors for communication skills. A study conducted on five medical schools in South California showed 94-96% accuracy of recording students’ performance by simulated patients.^18^ Use of checklist with guidebook and rigorous training improves the SPs’ skills to examine accurately. Alison et al did a study using year one medical students who were novice as examiners and were able to effectively differentiate between the communication skills of physicians and specialists. General physicians acquired higher scores in communication skills in comparison to specialists.^9^

The present study showed that SP gave higher scores in comparison to OBGYN and non-OBGYN faculty. Literature shows that SPs give higher ratings when students faced them directly along with facilitated nodding while listening and proper eye contact during the session.^16^ Secondly, SPs are not subject specialists, therefore, they mainly focus on communication skills. Adequate voice, tone and intonation give positive impression. Self-touching and unpurposive movements gave negative impression, this may indicate anxiety, tension and preoccupation of the student.^19^ These high scores raise some concern to use SPs for the decision of pass and fail in high stakes examinations.^20^ A research study investigated the accuracy and type of errors in recording. The SPs had gone through rigorous training and then utilized to assess the clinical performance of fourth year medical students. They divided the errors as commission (score given but student didn’t perform) and omission (student performed but score was not given). The commission errors were high 34.8% in comparison to omission errors 13.6%. This study endorsed the SP accuracy between 94-96% but whenever there is doubt it’s in favour of students.^18^ Another study investigated level of agreement among tutors and students with regard to cavity preparation. It was observed that discrepancies increased with level of task difficulty. However, after getting assessed and knowing the area where they didn’t perform well, improve the awareness and acceptance of mistakes.^21^

Non-experts are less critical in their marking, if they are not familiar with learning objectives. The students or novice rate themselves as more competent.^21^

### Limitation of the study

This study was limited to one university and the results may not be generalizable. There were all female residents in the present study, and all SPs used were also females. This study can be replicated in other contexts and institutions to see the generalizability of the results.

## CONCLUSION

Our study showed an excellent inter-rater reliability among SPs, subject and non-subject expert faculty, therefore both simulated patients and non OBGYN faculty can also be used confidently in formative assessments of communication skills. Non-OBGYN faculty can replace subject experts in high stakes examinations as well after training. On the other hand, the SPs can be engaged effectively and efficiently for teaching and learning of communication skills, as examiners for formative OSCEs along with provision of feedback to trainees. They may also be invited as examiners for summative OSCEs for communication skills with caution as they are lenient on marking with students.

## References

[1] Swing SR (2007). The ACGME outcome project:retrospective and prospective. Med Teach.

[2] (2002). Susan Swing,Hubert Dreyfus, Stuart Dreyfus. General Competencies And Accreditation In Graduate Medical Education. Health Aff.

[3] Makoul G (2003). Communication Skills Education in Medical School and Beyond. JAMA.

[4] Fong SF, Kramer K, Sakai DH, Kasuya RT, Ching N, Nishimura S (2015). Medical School Hotline:Liaison Committee on Medical Education Accreditation:Part II:The Graduation Objectives. Hawaii J Med Public Health.

[5] Chaudhry PZU CPSP integrates training, clinical service and research –BULLETIN College of Physicians and Surgeons Pakistan 2017.

[6] Chesser A, Reyes J, Woods NK, Williams K, Kraft R (2013). Reliability in patient-centered observations of family physicians. Fam Med.

[7] JCIA. oint Commission International Accreditation Standards for Hospitals (2017). Joint Commission International.

[8] Smith MB, Macieira TGR, Bumbach MD, Garbutt SJ, Citty SW, Stephen A (2018). The Use of Simulation to Teach Nursing Students and Clinicians Palliative Care and End-of-Life Communication:A systematic review. Am J Hosp Palliat Care.

[9] Vargovich AM, Sperry JA, Spero RA, Xiang J, Williams D (2016). Use of Checklists Teaches Communication Skills Utilized by Specialties. MedEdPublish.

[10] McKenzie CT, Tilashalski KR, Peterson DT, White ML (2017). Effectiveness of Standardized Patient Simulations in Teaching Clinical Communication Skills to Dental Students. J Dent Educ.

[11] Fatmi M, Hartling L, Hillier T, Campbell S, Oswald AE (2013). The effectiveness of team-based learning on learning outcomes in health professions education:BEME Guide No. 30. Med Teach.

[12] Changiz T, Jamshidian S, Entezari MH, Kassaian N (2014). Training and validation of standardized patients for evaluation of general practitioners'performance in management of obesity and overweight. Adv Biomed Res.

[13] Jabeen D (2013). Use of simulated patients for assessment of communication skills in undergraduate medical education in obstetrics and gynaecology. J Coll Physicians Surg Pak.

[14] Gowda SL, Bhandiwad A, Anupama NK (2016). Litigations in Obstetric and Gynecological Practice:Can it be prevented?A Probability to Possibility. J Obstet Gynaecol India.

[15] Nadeem N, Zafar AM, Zuberi RW, Ahmad MN (2012). Faculty and patient evaluations of radiology residents'communication and interpersonal skills. J Pak Med Assoc.

[16] McLaughlin K, Gregor L, Jones A, Coderre S (2006). Can standardized patients replace physicians as OSCE examiners?. BMC Med Educ.

[17] Holmboe, Stephen, Chung Jeff, Norcini John, Hawkins Richard E (2003). Construct Validity of the MiniClinical Evaluation Exercise (MiniCEX). Acad Med.

[18] Heine N, Garman K, Wallace P, Bartos R, Richards A (2003). An analysis of standardised patient checklist errors and their effect on student scores. Med Educ.

[19] Ishikawa H, Hashimoto H, Kinoshita M, Fujimori S, Shimizu T, Yano E (2006). Evaluating medical students'non-verbal communication during the objective structured clinical examination. Med Educ.

[20] Humphrey-Murto S, Smee S, Touchie C, Wood TJ, Blackmore DE (2005). A comparison of physician examiners and trained assessors in a high-stakes OSCE setting. Acad Med.

[21] San Diego JP, Newton T, Quinn BF, Cox MJ, Woolford MJ (2014). Levels of agreement between student and staff assessments of clinical skills in performing cavity preparation in artificial teeth. Eur J Dent Educ.

